# Engineered T Cell Therapy for Gynecologic Malignancies: Challenges and Opportunities

**DOI:** 10.3389/fimmu.2021.725330

**Published:** 2021-07-27

**Authors:** Yifan Xu, Jin Jiang, Yutong Wang, Wei Wang, Haokun Li, Wenyu Lai, Zhipeng Zhou, Wei Zhu, Zheng Xiang, Zhiming Wang, Zhe Zhu, Lingfeng Yu, Xiaolan Huang, Hua Zheng, Sha Wu

**Affiliations:** ^1^Microbiome Medicine Center, Department of Laboratory Medicine, Zhujiang Hospital, Southern Medical University, Guangzhou, China; ^2^Department of Immunology, School of Basic Medical Sciences, Southern Medical University, Guangzhou, China; ^3^Guangzhou Blood Center, Department of Blood Source Management, Guangzhou, China; ^4^Department of Obstetrics and Gynecology, The First Affiliated Hospital of Guangzhou Medical University, Guangzhou, China; ^5^Hepatology Unit and Department of Infectious Diseases, Nanfang Hospital, Southern Medical University, Guangzhou, China; ^6^Department of Paediatrics and Adolescent Medicine, Li Ka Shing Faculty of Medicine, University of Hong Kong, Hong Kong, China; ^7^State Key Laboratory of Esophageal Cancer Prevention & Treatment, Sino-British Research Center for Molecular Oncology, National Center for International Research in Cell and Gene Therapy, School of Basic Medical Sciences, Academy of Medical Sciences, Zhengzhou University, Zhengzhou, China; ^8^Huikezhe Biological Tech. Beijing, R&D Department, Beijing, China; ^9^School of Basic Medicine Science, Tianjin Medical University, Tianjin, China; ^10^Department of Cardiology, Nanfang Hospital, Southern Medical University, Guangzhou, China; ^11^National Demonstration Center for Experimental Education of Basic Medical Sciences, Southern Medical University, Guangzhou, China

**Keywords:** gynecologic malignancies, engineered T cells, CAR-T, TCR-T, adoptive T cell therapy, immunotherapy

## Abstract

Gynecologic malignancies, mainly including ovarian cancer, cervical cancer and endometrial cancer, are leading causes of death among women worldwide with high incidence and mortality rate. Recently, adoptive T cell therapy (ACT) using engineered T cells redirected by genes which encode for tumor-specific T cell receptors (TCRs) or chimeric antigen receptors (CARs) has demonstrated a delightful potency in B cell lymphoma treatment. Researches impelling ACT to be applied in treating solid tumors like gynecologic tumors are ongoing. This review summarizes the preclinical research and clinical application of engineered T cells therapy for gynecologic cancer in order to arouse new thoughts for remedies of this disease.

## Introduction

Gynecologic malignancies are serious threats to women’s health worldwide. Although traditional procedures like surgery, radiotherapy and chemotherapy have effectively decreased mortality, researchers are seeking new ideas and strategies to reduce the recurrence and metastasis of tumors, alleviate adverse drug reactions, as well as further improve the life quality of patients.

Adoptive T cell therapy (ACT) is one of the most powerful weapons among a wide range of approaches focusing on our immune system. The basic principle of this treatment refers to reinfusing autologous lymphocytes which are expanded, screened and modified *in vitro* to patients for tumor regression mediated by T cells. Early preclinical research successfully proved that with a genetically transferred synthetic receptor targeting antigen CD19, which is a broad marker commonly expressed by B cell lymphoma cells, reinfused autologous T cells could eliminate established B cell tumors in mice (1). Based on multiple tried-and-true basic experiments, clinical trials later showed prominent advantages of this kind of engineered T cells named chimeric antigen receptor T cells (CAR-Ts) in patients with hematological malignancies (2–5). Promoted by these significant achievements, adoptive T cell therapy has proved to be the potential adjuvant therapy for tumor treatment.

The application of natural tumor-infiltrating lymphocytes (TILs) obtained from suspension or fragments of the resected tumor is the earliest achievement of ACT. In 24^th^ May, 2019, a TIL product named LN-145 was granted as the breakthrough designation for cervical cancer (6), exhibiting remarkable objective response rate (ORR) and disease control rate (DCR) in treating cervical cancer (7). Although TILs have higher concentration of specific T cells comparing to peripheral T cells, the hostile tumor microenvironment attenuates the long-term survival of functional T cells, as TILs are sensitive to anergy, exhaustion and apoptosis. In addition, the gathering of TILs requires joint efforts of surgeons to obtain fresh tumor samples where effective lymphocytes could be extracted. Groundbreakingly, engineered T cells, including T cell receptor modified T cells (TCR-Ts) and CAR-Ts, currently have a promising advance in tumor immunotherapy since they could be genetically modified in structure to target specific tumor antigens or to express cytokines ameliorating immunosuppressive tumor microenvironment. Two CAR-T products have already been approved by the USA Food and Drug Administration (FDA) for refractory leukemia and lymphoma immunotherapy (8, 9).

**Figure 1:**
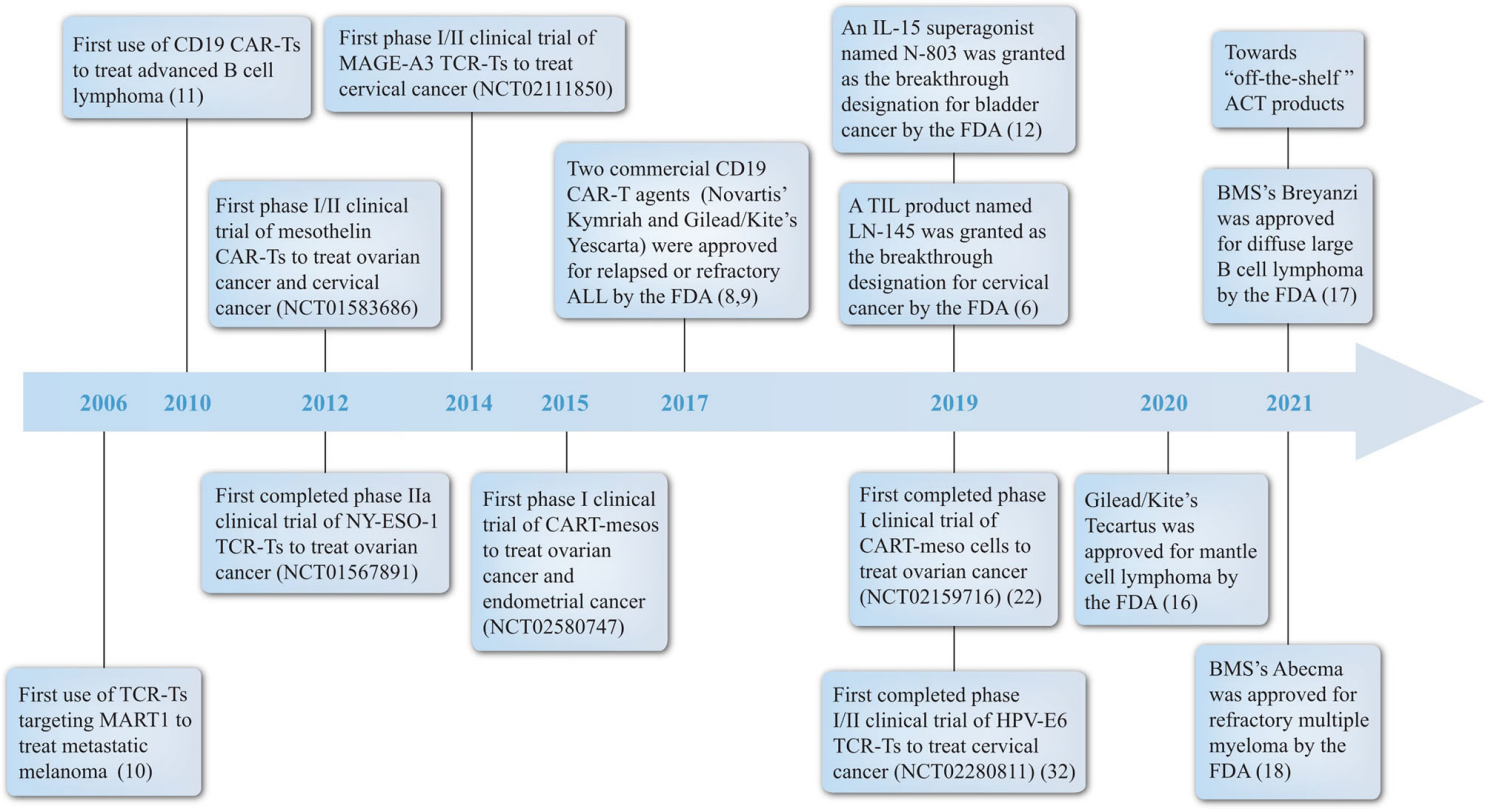
Milestones of ACT. A brief summary of some landmark achievements in ACT development history with a focus on engineered T cells for treating gynecologic malignancies from the year 2006 to 2021. Significant events include: (1) pioneer treatment of metastatic melanoma by TCR-T and B cell lymphoma by CAR-T; (2) the first or the fastest progressing clinical trial of engineered T cells in different gynecologic tumors; (3) the acknowledgement of CAR-T, TIL and IL-15 products by FDA. ACT, adoptive T cell therapy; BMS, Bristol-Myers Squibb; CAR, chimeric antigen receptor; FDA, the Food and Drug Administration; IL, interleukin; TCR, T cell receptor; TIL, tumor infiltrating lymphocyte.

In this review, we discuss the application of engineered T cells in gynecologic malignancies in preclinical and clinical trials, and explore further opportunities of implicating this therapy in clinical decision for gynecologic oncology. A brief timeline of milestones associated with this field is arranged (**Figure 1**). Pioneer clinical application of engineered T cells, critical clinical trials carried out for gynecologic cancers and commercial CAR-T agents and related synergist approved by the FDA are included (10–12).

## Engineered T cells

Based on the gene editing technology, engineered peripheral T cells with specific antigen binding receptors like TCRs or CARs could further facilitate ACT progress compared with TILs. These two therapies have different mechanisms and efficiency preference for treating distinct tumors. Currently, mainstream cell preparation methods include the following steps: (1) obtaining frozen apheresis white blood cell (WBC) product from patients; (2) the selection and enrichment of T cells by corresponding selection beads; (3) activation of T cells *via* addition of stimulating cytokines like interleukin (IL) 2 and beads like anti-CD3/CD28 beads; (4) transduction of target CAR or TCR genes through lentiviral, retroviral vectors or transposase systems and so on; (5) expanding the number of T cells *in vitro*; (6) cryopreservation.

### T Cell Receptor Modified T Cells (TCR-Ts) Therapy

TCRs are specific receptors on the surface of T cells capable of recognizing peptide major histocompatibility complex (pMHC) formed by peptide antigens presented by the MHC on tumor or antigen presenting cells. The killing ability of CD8+ T cells depends on the specific identification of cleaved peptide chains bound to class I human leukocyte antigen (HLA) by TCRs, therefore it is noteworthy that the function of TCRs only works in HLA-appropriate patients. T cell sources derived from individuals or humanized mice with matched HLA alleles and sophisticated techniques are required for the personalized production of TCRs. The alpha and beta chain pair of TCRs can be genetically modified to target tumor antigens and thus T cells transfected with these new TCRs can specifically recognize and eliminate cancer cells. Recently, a non-virus solution using the Sleeping Beauty (SB) transposons system to target unique neoantigens was described (13), which exhibited advantages with lower price and risk of random insertional mutagenesis.

Compared with the antibody-binding-like principle of CAR-Ts, TCR-Ts can recognize target antigens more extensively since they not only identify cell membrane antigens but also intracellular tumor antigens presented by pMHC, inducing a more orderly and durable immunological synapse formation process. Particularly, the targeting of almost 90% solid tumors relies on tumor specific antigens (TSAs) inside tumor cells, while surface antigens are often tumor associated antigens (TAAs) which can also be expressed by normal tissues to affect their function. Besides, TCR-Ts follow the natural signaling pathway to maintain their original regulatory mechanism, being more sensitive to low-copy antigens than CAR-Ts. Consequently, the potential of TCR-Ts dramatically outweighs CAR-Ts in treating solid tumors (14). However, the utility of TCR-Ts in treating solid tumors is progressing slowly. Currently, there is no market approval for any TCR-T products. Several clinical trials are still ongoing.

### Chimeric Antigen Receptor T Cells (CAR-Ts) Therapy

**Figure 2:**
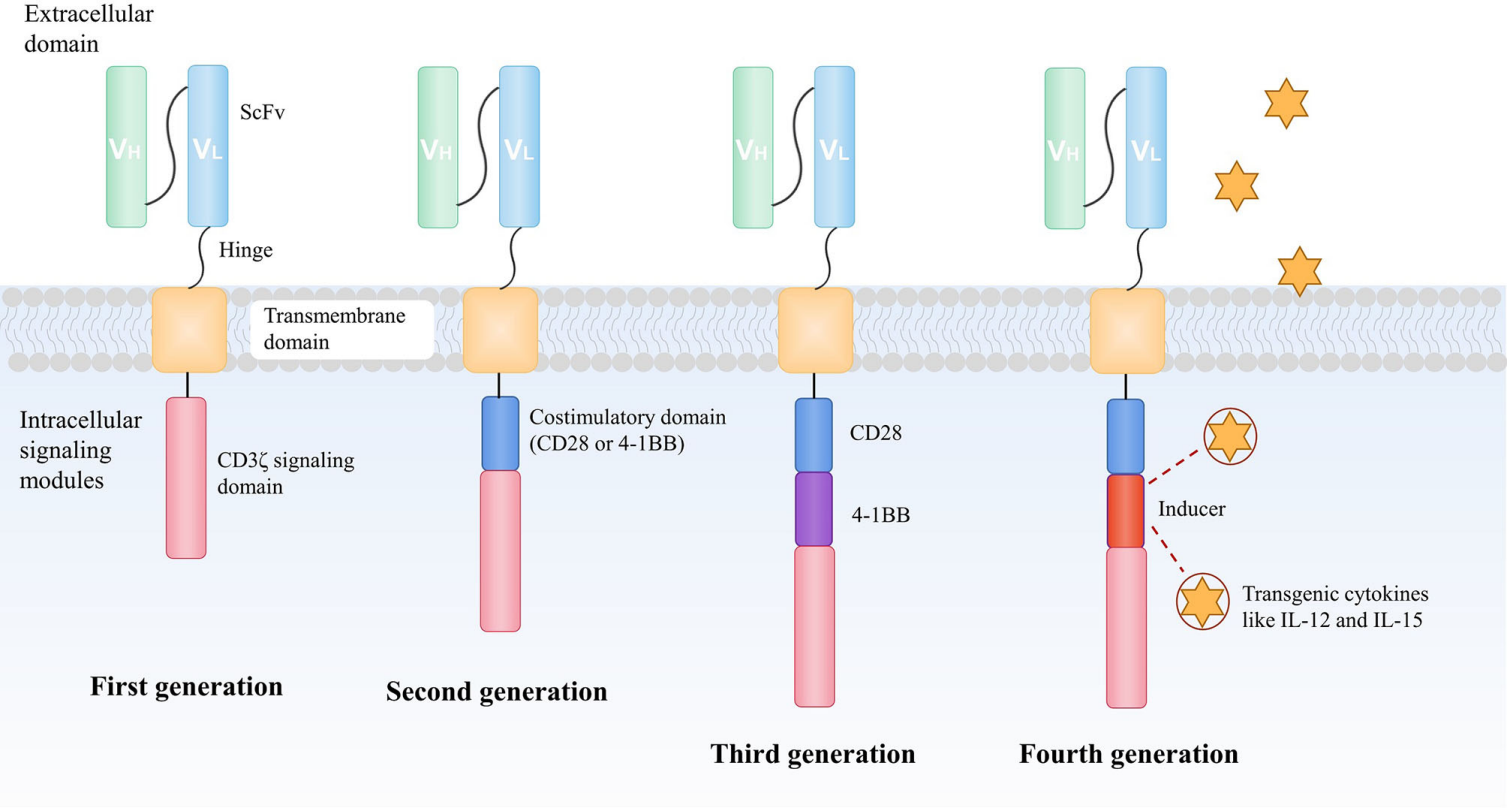
The development of CAR construction. A CAR is composed of an extracellular antigen-binding domain, most of which is an antibody–derived scFV, a transmembrane domain and an intracellular signaling domain of the TCR CD3ζchain to activate T cells. To enhance the antitumor ability of CAR-T, the design of CARs has evolved over recent years. The second generation of CAR consists of an additional co-stimulatory domain, usually CD28 or 4-1BB (CD137) moieties to improve the capacity of persistence and proliferation of T cells. An extra co-stimulatory domain (CD28 and 4-1BB or TLR2) is added in the third-generation of CAR to further augment the efficacy of infused CAR-T cells. In the fourth generation of CAR, the intracellular segment of the cytokine receptor is also added to the CAR, which effectively promotes the expansion of T cells. CAR, chimeric antigen receptor; scFv, antibody-derived single-chain variable fragment; TCR, T cell receptor; TLR, toll-like receptor.

The most obvious character of CAR-T cells in contrast to TCR-T cells is that CARs can directly bind antigens in an MHC-independent fashion, therefore they are potentially able to detect most of the surface-expressing targets in patients who have various HLA types. This is particularly important for immunotherapy because tumor cells losing MHC-associated antigens are probable to escape immune surveillance. A CAR is composed of an extracellular antigen-binding domain, most of which is an antibody–derived single-chain variable fragment (scFV), a transmembrane domain and an intracellular signaling domain of the TCR CD3ζ chain to activate T cells (15). The consisting improvements of CAR-T include the introduction of an additional co-stimulatory molecular CD28 or 4-1BB (CD137) intracellular domain (16), and inducers for transgenic cytokines like IL-12 and IL-15 (17) (**Figure 2**).

The landmark of CAR-T therapy is the commercial CD19 specific CAR-T approved by the FDA for relapsed or refractory acute lymphocytic leukemia (ALL). Two commercial agents, tisagenlecleucel (Kymriah, Novartis) (9) and axicabtagene ciloleucel (Yescarta, Kite Pharma) (8) were acknowledged in 2017. After this, brexucabtagene autoleucel (Tecartus, Kite Pharma) (18), lisocabtagene maraleucel (Breyanzi, Bristol-Myers Squibb) (19) and idecabtagene vicleucel (Abecma, Bristol-Myers Squibb) (20) were approved successively by the FDA for marketing, further promoting the clinical implement of CAR-T therapy in hematological malignancies. Among these agents, only Abecma targets B cell maturation antigen (BCMA), others continue to focus on CD19.

## Studies of Engineered T Cells in Common Malignant Gynecologic Tumors

Unlike the popularity of CAR-T therapy in hematological malignancies, studies for broader swaths in the field of gynecologic tumors are still in the bud. Antigen selection is crucial in deciding treatment programs which lead to TCR-T or CAR-T therapy and the treatment efficiency. Where the antigen is expressed at the cell and tissue level should be the first consideration by high-throughput, ultra-sensitive mass spectrometry and other means when ACT is carried out. Improvements could be reflected in the optimization of antigen selection for patients with different types of gynecological tumors in the future.

### Ovarian Cancer

Ovarian cancer significantly jeopardizes the health of women with high lethality. With advanced surgical treatment and systematic care, the five-year relative survival rate of patients is slightly promoted, but still less than 50% (21).

Armed with the knowledge that the melanoma-associated antigen 4 (MAGE-A4) and the New York esophageal squamous cell carcinoma 1 (NY-ESO-1) are commonly expressed by ovarian cancer cells (26.4% and 3.6% respectively) (22), TCR-T products targeting these two ideal antigens have been designed and applied in clinical research. MAGE-A4^c1032^T cells are used in HLA-A*02:01 (A2+) patients with MAGE-A4 positive tumors including ovarian cancer in an ongoing phase I multi-tumor study (NCT03132922). In cohort 3/expansion (28 patients), 7 patients with synovial sarcoma had partial response (PR), 11 patients had stable disease (SD), 5 patients had progressive disease (PD) and the remaining 5 were non-evaluable. MAGE-A4 specific TCR-T exhibited therapeutic potential and manageable adverse effects at a dose range of (1.2~10) ×10^9^ (23). In further research, a CD8α co-receptor was introduced into CD4+ T cells alongside the engineered TCR (ADP-A2M4CD8). These modified CD4+ T cells could in turn elevate the cytotoxicity and expansion of effector CD8+ T cells (24). NY-ESO-1 is the most broadly researched antigen with a panel of phase I/II clinical studies ongoing (NCT01567891, NCT03159585, NCT03691376, NCT03017131, NCT02869217). TBI-1301 is a cell product which is genetically modified to express NY-ESO-1 specific TCR. Butler et al. conducted a phase Ib clinical trial using TBI-1301 to treat HLA-A*02:01+ or A*02:06+ patients with NY-ESO-1+ solid cancers (NCT02869217). The ovarian patient had SD for 4.7 months and the standard dose infused was 5×10^9^ (25). Another study used affinity enhanced autologous NY-ESO-1^c259^T cells for treating HLA-A*02:01, *02:05, or *02:06 positive recurrent ovarian cancer (NCT01567891). However, so far, no objective tumor response has been recorded for 6 patients who completed the research.

Mesothelin (Msln) is another frontier antigen for ovarian cancer. Anderson et al. conducted a preclinical experiment with Msln specific TCR_1045_ T cells. These T cells exhibited tumor cytotoxicity both in ID8_VEGF_ ovarian cancer cells and in murine model, but the function was on the wane within 21 days. To enhance the antitumor activity, engineered T cells were repeatedly infused to mice and a maintained effect was seen. The time to progression (TTP) for TCR_1045_ plus an irradiated peptide-pulsed splenocyte vaccine was longer than that of using T cells alone or no-treatment group (112 days, 91 days, 77 days) (26).

Findings for targeting mesothelin in CAR-T therapy are also of note. Haas et al. enrolled five patients with mesothelin expressing recurrent ovarian cancer in a phase I study (NCT02159716). The most significant result was seen in ovarian cancer among multiple mesothelin+ tumors involved. Patients received lentiviral transduced CART-meso cells with different doses: two were infused with (1~3)×10^8^/m^2^ cells, and three were infused with (1~3)×10^7^/m^2^ cells, both groups were evaluated as SD for 28 days. Although the function of tumor control was observed, these antitumor responses were transient and limited (27). A case of patient with refractory epithelial ovarian cancer after chemotherapy was reported recently. The patient received two infusions of CAR-Ts encoded by genes specific for mesothelin and the immune checkpoint inhibitors. An antiangiogenic drug inhibiting vascular endothelial growth factor receptor (VEGFR)-2 named apatinib was included in the treatment. The follow-up assessment showed partial response with attenuated diameter of liver metastatic nodules and a 17-month survival (NCT03615313). Only slight adverse reactions were observed (28). Zhao et al. revealed that humanized (hu) CD19 specific CAR had 6-fold higher affinity compared with murine CAR (29). Murine CAR has different structure domains which tend to trigger adaptive immunity. Once immune recognition of murine scFv is established, the therapeutic effect would be considerably subdued. Improved strategy employing huCART-meso cells to treat cancers commonly express mesothelin is now recruiting candidates (NCT03054298). A research using the fourth generation CAR-Ts for refractory or relapsed ovarian cancer has just been initiated with outcomes remaining to be seen (NCT03814447).

Mucin 16 (MUC16) is a glycosylated mucin widely expressed in ovarian cancer, serving as a promising target for CAR-T therapy. A phase I clinical trial is ongoing with MUC-16ecto CAR-T cells to treat recurrent ovarian cancer (NCT02498912). 5 dose levels are planned for the assessment of the maximum tolerated dose (3×10^5^, 1×10^6^, 3×10^6^, 1×10^7^, 3×10^7^). Furthermore, these CAR-T cells are modified to secrete IL-12, which could improve T cell persistence and overcome various inhibitions from the tumor microenvironment (30). Nectin is a class of cell adhesion molecule which belongs to the Ca^2+^-independent immunoglobulin superfamily proteins. Nectin-4 is expressed in various organs during fetal development but barely expressed in adults other than placenta. In ovarian tumor tissues, nectin-4 is overexpressed and plays a key role in tumor cell adhesion, migration, aggregation and proliferation (31). Currently there is a phase I clinical trial using the CAR-T, which involves in various costimulatory domains and cytokines (IL-7 and CCL19, or IL-12) to treat nectin-4 positive ovarian cancer (NCT03932565). Recently, Garcia et al. provided evidence that T cells with CAR targeting Müllerian inhibiting substance type 2 receptor (MISIIR) were tumoricidal both *in vitro* and *in vivo* and no reaction was reported to normal primary human cells. Especially, MISIIR specific CAR-Ts lysed multiple human ovarian and other gynecologic cancer cells, showing potency in treating gynecologic malignancies in the clinic (32).

PRGN-3005 UltraCAR-T was engineered to express MUC-16, membrane bound IL-15 (mbIL-15) to promote persistence of T cells and the kill switch to ensure safety simultaneously. It was applied in a phase I clinical trial for patients with advanced and recurrent platinum-resistant ovarian cancer in 2019 (NCT03907527). This is a seminal gene and cellular therapy which owns a non-viral multigenetic transfer patent to produce UltraCAR-T cells without the need for *in vitro* proliferation, thus shortening the waiting period from several weeks to one day. This landmark study has the potential to allow the therapy accessible to common patients by reducing costs. It also holds promise for subverting the current pattern of CAR-T cell therapy by regulating the immune system and tumor targeting in a more precise fashion (33).

Studies have demonstrated that the combination of ACT and immune checkpoint inhibitor (Pembrolizumab and Nivolumab) can fight against T cell exhaustion induced by immune checkpoints and augment the antitumor activity in the treatment of advanced, recurrent or metastatic programmed cell death protein ligand 1 (PD-L1) expressing gynecologic malignancies (34). Accordingly, a programmed cell death protein 1 (PD-1) gene-knocked out transferred T cell product has been promoted recently *via* gene editing technology (CRISPR-Cas9, lentivirus technology, etc.). A phase I clinical study evaluating the safety and efficiency of PD-1 gene-knocked out CART-meso cells for treating mesothelin positive multiple solid tumors is currently ongoing (NCT03747965). A clinical trial of advanced refractory ovarian cancer using αPD-1 CART-meso cell therapy combined with apatinib was also observed with potential therapeutic effect, which is detailed mentioned above (NCT03615313).

### Cervical Cancer

Cervical cancer is one of the most common gynecologic malignancies bothering middle-aged women, especially in developing countries. Although the incidence and mortality of cervical cancer have declined in recent years, the morbidity crowd tends to be younger, which is still worthy of vigilance (35).

The infection with high-risk human papillomavirus (HR-HPV) is a noted driver for the development of nearly all cervical cancers. E6 and E7 oncoproteins are highly expressed by HPV+ cervical cancer cells, becoming attractive therapeutic targets for engineered T cells. Preclinical research revealed that HPV-16 E6 (36)/E7 (37) specific TCR-Ts could detect and kill HLA-A2+ HPV-16+ tumor cells *in vitro* without cross-reactivity against human self-peptides. The antitumor avidity of E7 TCR-Ts against cervical cancer was also verified in a murine model.

A phase I/II study of HLA-A2 restricted E6 TCR-Ts for HPV-associated cancers (NCT02280811) was reported by Doran et al. Other interventions include common conditioning regimen, and systemic aldesleukin. Among 6 cervical cancer patients, 2 of them displayed SD, one for 6 months, another for 4 months. The percentage of E6 T cells in infused cells (range from (1~170)×10^9^) were 51% and 71% respectively. In the phase I portion, no severe adverse effects were observed (38). A first-in-human, phase I clinical trial of HLA-A2 restricted E7 TCR-Ts to treat patients with metastatic HPV-16+ cancers has just uploaded its report (NCT02858310). Two in five patients with cervical cancer displayed PR for 8 months and 3 months, with T cell portion in infused cells (range from (1~107)×10^9^) being 97% and 96%, respectively. One patient had SD for 3 months, and no response was observed in the remaining two patients. Researchers also proposed that genetic defects in the key elements of the antigen presentation and interferon response were responsible for treatment resistance of ACT (39). Some patients combined the PD-1 blockade therapy to improve T cell infiltration. In trial NCT03578406, five patients were treated with E6 TCR-T monotherapy: two of them received 5×10^6^/kg dose and three received 1×10^7^/kg dose. 28 days later, three patients had SD, one patient had PD, one patient was loss to follow-up. In another arm, two patients were infused with 5×10^6^/kg and 1×10^7^/kg of anti-PD-1 TCR-Ts respectively. The patient with lower dose was assessed as SD at both day 28 and month 2 post-infusion, showing promising efficiency for combining engineered T cell therapy with immune checkpoint inhibitor for cervical cancer patients (40).

New therapeutic targets of CAR products have been widely expanded *via* several preclinical researches which have progressed to the stage of animal experiments. CD47 specific CAR-Ts were proved to effectively kill ovarian, pancreatic, and cervical cancer cell lines and retard pancreatic tumor growth in mice (41). Recently, the antitumor efficiency of CART-meso cells was illustrated in SiHa cells *in vitro* by elevated levels of IL-4、IL-2、IL-5、tumor necrosis factor (TNF) α and interferon (IFN) γ secretion. The capacity in tumor control sustained for about 1 week *in vivo.* Better results were obtained following the second injection of T cells (42). Positive responses were also observed in Hela, SiHa, ME-180 and C-33A cell lines and in murine models through natural killer group 2D (NKG2D)/NKG2D-ligand pathway (43).

Currently, a phase I/II study of CART-meso cells in treating metastatic cancers including cervical cancer and ovarian cancer has been terminated with only one patient assessed as SD for > 3.5 months (NCT01583686). There is an ongoing phase I/II clinical trial using CARs targeting antigens such as GD2, prostate specific membrane antigen (PSMA), MUC-1, mesothelin or other markers positive to cervical cancer (NCT03356795). CD22 is often selected as the target for B cell malignancy. Recently, a phase I study employed CD22 specific CAR-Ts to treat solid tumors, including cervical cancer (NCT04556669). They also introduced the anti-PD-L1 monoclonal antibody to the CAR structure. More clinical evidence regarding the efficiency of CAR-T therapy for cervical cancer is required.

### Endometrial Cancer

Endometrial cancer (EC) is the sixth most common cancer in women, and this ranking may rise especially in western countries (44). Although the 5-year survival rate of patients in the early stage is 95%, it would sharply decrease to 16% to patients with advanced or recurrent metastatic tumors (45).

There are not enough reports for the clinical assessment of ACT in EC until now. Only one patient treated with 5×10^9^ TBI-1301 showed SD for 3.6 months without cytokine release syndrome (CRS) in a phase Ib clinical trial which has been mentioned above (NCT02869217). On 13 Nov 2020, a phase I/II clinical trial has just been initiated using CAR-Ts targeting alkaline phosphatase, placental (ALPP) for endometrial cancer and ovarian cancer (NCT04627740). The primary outcome measures related adverse events and the secondary outcome measures ORR, progression-free survival (PFS) and the number of transferred T cells.

### Vulvar Squamous Cell Carcinoma

High-grade squamous intraepithelial lesion (HSIL) is a precancerous lesion of vulvar squamous cell carcinoma (VSCC) caused by HPV infection (46). The risk of cancer development can be reduced by treating HSIL. TCR-Ts targeting HPV-16 E6 protein thus provide a therapeutic window for HSIL to further prevent VSCC. A related phase I clinical trial was closed due to the lack of perceived clinical activity observed in the study (NCT03197025). A phase II study of HPV-16 E7 TCR-Ts for treating HSIL was also terminated without concrete results (NCT03937791). In a clinical study of E7 specific TCR-Ts mentioned above, vulvar diseases are included (NCT02858310).

## The Challenges With Engineered T Cells in Gynecologic Oncology

Several challenges become apparent when it comes to the promotion of engineered T cells. The major concern with this therapy is the severe adverse effect. TAAs can also be expressed by normal tissues, causing undesired on-target/off-tumor toxicity. CD19 CAR-Ts could induce the deficiency of normal CD19+B cells and cause weakened immunity. Besides, some TCRs or CARs are not specific to target antigen, but cross-react to other self-antigens. Taking MAGE-A3 specific TCR-Ts as an example, in previous studies, there were fatal events associated with injury in MAGE-A13 expressing tissues like the nervous system (47) and titin of cardiac cells (48, 49). MAGE-A13 was marginally expressed but unexpected and deadly destructive. Antigen selection is the first consideration in designing an ACT protocol. It is critical to choose ideal antigens that are tumor-specific, carcinogenic and immunogenic in order to strengthen the antitumor efficiency and reduce related toxicity simultaneously. In clinical trials using TCR-Ts to treat gynecologic malignancies, the target antigens involve: HPV16-E6/E7, NY-ESO-1, MAGE-A3, MAGE-A4, mesothelin. Antigens used as CAR-T therapeutic targets include: mesothelin, CD70, CD22, CD133, GD2, PSMA, MUC1, MUC16, human epidermal growth factor receptor 2 (HER-2), nectin-4, anti-alpha folate receptor (FR-α), ALPP, B7-H3, TnMUC1 (**Table 1**). In recent years, neoantigens have also emerged as a potential therapeutic option for gynecologic tumors since they are induced by somatic point mutations in tumor cells instead of co-expression with normal tissues. Matsuda et al. have successfully generated 3 neoantigen-specific TCRs through whole-exome sequencing (WES) of 7 ovarian tumors and the induction of peripheral blood mononuclear cells (PBMCs) isolated from healthy donors. These T cells could recognize their corresponding neoantigens although cross-reactivity to the wild-type peptide was observed in one of them (50). As an infant in the field of immunotherapy, it warrants further investigation whether these neoantigens will continue to be stably expressed by tumor cells.

**Table 1:** Clinical trials of engineered T cells in gynecologic cancer immunotherapy (www.clinicaltrails.com).

Cancer	Type	antigen	Stage and Result	Host	NCT
Ovarian cancer	TCR-T	MAGE-A4	Phase I (recruiting)	University of Miami, USA	NCT03132922
			7 pts had PR, 11 had SD, 5 had PD		
	TCR-T	NY-ESO-1	Phase IIa (completed with results)	City of Hope National Medical Center, USA	NCT01567891
			No objective effects have been reported		
	TCR-T	NY-ESO-1	Phase I (completed without results)	Zhujiang Hospital of Southern Mediacal University, China	NCT03159585
	TCR-T	NY-ESO-1	Phase I (recruiting)	Roswell Park Cancer Institute, USA	NCT03691376
	TCR-T	NY-ESO-1	Phase I (active, not recruiting)	Roswell Park Cancer Institute, USA	NCT03017131
	TCR-T	NY-ESO-1	Phase Ib (recruiting)	Princess Margaret Cancer Centre, Canada	NCT02869217
			One patient had SD for 4.7m with grade 2 CRS		
	TCR-T	NY-ESO-1	Phase I (unknown)	Shenzhen Second People’s Hospital, China	NCT02457650
	TCR-T	Neoantigen	Phase II (suspended)	National Institutes of Health Clinical Center, USA	NCT04102436
	TCR-T	Neoantigen	Phase II (suspended)	National Institutes of Health Clinical Center, USA	NCT03412877
	CAR-T	Mesothelin	Phase I (completed with results)	Abramson Cancer Center of the University of Pennsylvania, USA	NCT02159716
			Five patients had SD for 28 days		
	Hu CAR-T	Mesothelin	Phase I (recruiting)	University of Pennsylvania, USA	NCT03054298
	CAR-T	Mesothelin	Early Phase I (recruiting)	Shanghai 6th People’s Hospital, China	NCT03814447
	CAR-T	Mesothelin	Phase I (terminated)	National Institutes of Health Clinical Center, USA	NCT01583686
			Only one patient had SD for > 3.5m		
	CAR-T	Mesothelin	Phase I/II (recruiting)	The Second Affiliated hospital of Zhejiang University School of Medicine, China	NCT03916679
	CAR-T	Mesothelin	Early Phase I (recruiting)	The Second Affiliated hospital of Zhejiang University School of Medicine, China	NCT03799913
	CAR-T	Mesothelin	Phase I (recruiting)	Shanghai East Hospital, China	NCT04562298
	CAR-T	Mesothelin	Phase I (Active, not recruiting)	National Cancer Institute, USA	NCT03608618
	CAR-T	Mesothelin	Phase I (unknown)	Biotherapeutic Department and Pediatrics Department of Chinese PLA General Hospital	NCT02580747
	αPD1-CAR T	Mesothelin	Early Phase I (recruiting)	Shanghai 10th people’s Hospital, China	NCT04503980
	αPD1-CAR T	Mesothelin	Phase I/II (recruiting)	Shanghai Cell Therapy Research Institute.	NCT03615313
	CAR-T	MUC16	Phase I (active, not recruiting)	Memorial Sloan Kettering Cancer Center, USA	NCT02498912
	CAR-T	Nectin4/FAP	Phase I (recruiting)	The Sixth Affiliated Hospital of Wenzhou Medical University, China	NCT03932565
	UltraCAR-T	MUC16	Phase I (recruiting)	Fred Hutch/University of Washington Cancer Consortium, USA	NCT03907527
	CAR-T	B7-H3	Phase I (not yet recruiting)	Lineberger Comprehensive Cancer Center, USA	NCT04670068
	CAR-T	ALPP	Phase I/II (not yet recruiting)	Xinqiao Hospital of Chongqing, China	NCT04627740
	CAR-T	FRα	Phase I (recruiting)	University of Pennsylvania Health System, USA	NCT03585764
	CAR-T	CD133	Phase I (completed without results)	Biotherapeutic Department and Pediatrics Department of Chinese PLA General Hospital	NCT02541370
	CAR-T	HER-2	Phase I (recruiting)	Zhongshan Hospital Affiliated to Fudan University, China	NCT04511871
	CAR-T	HER-2	Phase I/II (withdrawn)	Southwest Hospital of Third Millitary Medical University, China	NCT02713984
	CAR-T	CD70	Phase I/II (suspended)	National Institutes of Health Clinical Center, USA	NCT02830724
	CAR-T	TnMUC1	Phase I (recruiting)	The Angeles Clinic and Research Institute, USA	NCT04025216
Cervical cancer	TCR-T	HPV-E6	Phase I/II (completed with results)	National Institutes of Health Clinical Center, USA	NCT02280811
			One patient had SD for 6m, one had SD for 4m		
	αPD1-TCR T	HPV-E6	Phase I (recruiting)	Qingzhu Jia, Chongqing, China	NCT03578406
			Enhanced SD in combination with anti-PD-1 therapy		
	TCR-T	HPV-E7	Phase I/II (recruiting)	National Institutes of Health Clinical Center, USA	NCT02858310
	TCR-T	HPV-E7	Early Phase I (suspended)	National Institutes of Health Clinical Center, USA	NCT04476251
	TCR-T	HPV-E7	Phase I (withdrawn)	National Institutes of Health Clinical Center, USA	NCT04411134
	TCR-CD4+ T	MAGE-A3	Phase I/II (active, not recruiting)	National Institutes of Health Clinical Center, USA	NCT02111850
			One patient had CR for > 29m		
	TCR-T	MAGE-A3	Phase I/II (terminated)	National Institutes of Health Clinical Cente, USA	NCT02153905
			One patient had PR after 6w and 12w		
	CAR-T	Mesothelin	Phase I (terminated)	National Institutes of Health Clinical Center, USA	NCT01583686
			Only one patient had SD for > 3.5m		
	αPD1-CAR-T	CD22	Phase I (recruiting)	Fourth Hospital of Hebei Medical University, China	NCT04556669
	CAR-T	GD2, PSMA, MUC1, Msln	Phase I/II (recruiting)	Shenzhen Geno-immune Medical Institute, China	NCT03356795
Endometrial cancer	CAR-T	Mesothelin	Phase I (unknown)	Biotherapeutic Department and Pediatrics Department of Chinese PLA General Hospital	NCT02580747
	CAR-T	ALPP	Phase I/II (not yet recruiting)	Xinqiao Hospital of Chongqing, China	NCT04627740
Vulvar squamous cell carcinoma	TCR-T	HPV-E6	Phase I (terminated)	National Institutes of Health Clinical Center, USA	NCT03197025
	TCR-T	HPV-E7	Phase II (terminated)	National Institutes of Health Clinical Center, USA	NCT03937791
	TCR-T	HPV-E7	Phase I/II (recruiting)	National Institutes of Health Clinical Center, USA	NCT02858310

ALPP, alkaline phosphatase, placental; CAR, chimeric antigen receptor; CR, complete response; CRS, cytokine release syndrome; FAP, fibroblast activation protein; FRα, anti-alpha folate receptor; HER-2, human epidermal growth factor receptor 2; HPV, human papillomavirus; MAGE-A, melanoma-associated antigen; Msln, mesothelin; MUC16, mucin 16; NY-ESO-1, New York esophageal squamous cell carcinoma 1; PD, progressive disease; PD-1, programmed cell death protein 1; PR, partial response; PSMA, prostate specific membrane antigen; SD, stable disease; TCR, T cell receptor.

CRS is another common threat particularly for CAR-T treatment. The excessive stress reaction of immune system would release superabundant cytokines such as TNF-α、IL-1、IL-6、IL-12、IFN-α、IFN-γ, leading to systemic inflammatory response syndrome (SIRS) and multiple organ failure. Grade 3 and 4 CRS can be life-threatening. In a multicenter clinical trial using CD19 CAR-Ts to treat refractory diffuse large B-cell lymphoma, 20% patients had grade ≥3 CRS events. More seriously, a rare case of fulminant haemophagocytic lymphohistiocytosis was reported (51). In another trial of CD19 CAR-Ts treating refractory ALL, 3 cases of death induced by refractory CRS were reported (52). Management methods of CRS include: monoclonal antibodies against IL-6 (siltuximab, clazakizumab) and its receptor (tocilizumab), IL-1 receptor (anakinra), glucocorticoids, alemtuzumab and etc (53). In trial NCT02869217, the patient with ovarian cancer had grade 2 CRS which required tocilizumab to manage.

Tumor heterogeneity is reflected in different sites of the same tumor or its recurrent lesion, being responsible for antigen escape. The loss of target antigen after ACT represents a key mechanism in the recurrence of tumor. Unfavorable feedback has been obtained from CD19-negative relapses. In up to 60% patients with refractory ALL, relapses after receiving CD19 CAR-T therapy could happen due to the loss of CD19 antigen. Once the antigen load is insufficient to activate immunoreaction, patients would become resistant to CAR-T therapy. Efforts were made to overcome this obstacle through establishing a dual CAR-T which could combine an additional antigen like CD123, a stem cell marker expressed in CD19-negative relapses, to prevent possible antigen loss (54).

The immunosuppressive microenvironment is a contributing factor to the proliferation, metastasis and drug resistance of gynecologic tumor cells. Particularly, abdominal cavity metastasis is a common pathological feature of ovarian cancer, and the formation of ascitic fluid provides a favorable microenvironment for affecting tumor growth and invasiveness. It promotes vascular and lymphangiogenesis in tumor tissues and enables tumor cells to evade immune surveillance *via* several pathways: (1) offering ligands for immune checkpoint proteins, such as PD-1 and cytotoxic T lymphocyte associate protein-4 (CTLA-4); (2) providing an immune suppressive setting through cytokines such as IL-10, IL-6, TGF-β vascular endothelial growth factor (VEGF) and so on, extracellular matrix components like matrix metalloproteinases (MMPs) or suppressive cells such as myeloid-derived suppressor cells (MDSCs) and regulatory T cells (Tregs); (3) interaction with multiple active substances in stromal cells, such as tumor-associated macrophages (TAMs), cancer‐associated fibroblasts (CAFs), and endothelial cells; (4) creating a physically and chemically hostile metabolic environment that is hypoxia, glucose-deficient, acidic, full of indolamine-1-oxidase and arginase (55).

The application of CAR-T therapy has long been constrained with unsatisfactory results in solid tumors including gynecologic tumors. A major hindrance for the broader use of CAR-Ts is attributed to the resistance of tumor microenvironment. Researchers found that by expressing IL-7 and CCL19 in CAR-Ts in mice, the immune cell infiltration in tumor tissues increased, thus reinforcing antitumor effects (56). In addition, chemokines e.g. CCR2b (57) and CCR4 (58) are factors affecting the progression and metastasis of tumor. Conversely, they can also facilitate the tumor infiltration of CAR-Ts when co-expressed with T lymphocytes. Although attempts in the combination of immune checkpoint blockades and ACT seem to make reversing the inhibitory microenvironment a reality, this strategy is still flawed due to neglect of the systemic network comprised of multiple immune suppressive mechanisms. A more concentrated attack on solid tumors is to use lipid nanoparticles to ferry immune-modulatory agents that are pertinently combined into components of tumor microenvironment. Compared with monotherapy, the level of TAMs, MDSCs and Tregs all reduced (9.4-fold, 4.6-fold, 4.8-fold), and the concentration of antitumor cells like CD8+ T cells and invariant natural killer T cells (iNKTs) increased (6.2-fold, 29.8-fold) (59). It seems to be a promising method with less cost, labor and fewer adverse effects.

The transient persistence of transferred T cells also makes it challenging to achieve optimal clinical results. Increasing the number of long-term memory T cells is a feasible way in obtaining sustained immunity. Stem memory T cells (Tscm) are superiorly potential in self-renewal, proliferation and long-last existence compared with T cells in other stages (60). Exploring approaches to induce Tscm-like T cells has been a hot spot of tumor immunology in recent years. Productive methods include cancer vaccines with regulated TCR signaling (61), co-culture with cytokines like IL-7, IL-15, IL-21 (62), and the addition of co-stimulation domains (63).

## The Future of Engineered T Cells in the Field of Gynecologic Tumors

An essential contributing factor for the broader application of engineered ACT technology is a systematically manufactured process. The whole process should be strictly controlled with quality testing to obviate contamination and satisfy clinical demand. Although multiple CAR-T agents have been permitted into the market, the preparation of T cells before treatment is still performed in a personalized pattern, which is time-consuming for 12 days in average with small scale (64). The protocol is now embracing a more automatic and universal fashion called ‘off-the-shelf’ ACT manufacture using allogenic T cells that are modified to be mildly immunoreactive to the host (65). Importantly, the depletion of allogeneic TCR, class I HLA molecule of donor T cells with CRISPR-Cas9 system would make ‘off-the-shelf’ CAR-Ts come true by reducing the risk of graft-versus-host disease (GVHD) (66).

The efficiency of engineered T cells in treating gynecologic tumors is currently not fully supported by sufficient clinical data and warrants further attempts in the clinical setting. Efforts to break barriers discussed above such as antigen selection, toxicities, the immune-unfavorable microenvironment in gynecologic tumors, the persistence of infused cells are making headway. Future investigation should provide update on these topics: (1) carrying forward clinical and preclinical trials; (2) more appropriate antigen binding sites; (3) how to break barriers to produce engineered T cell in a larger scale without toxicity; (4) how to maintain the cytotoxicity of engineered T cells in the tumor microenvironment; (5) synergistic treatment with immune checkpoint inhibitors or other substances. With further work to be done and deeper understanding of ACT, it would present a potential treatment for gynecologic oncology.

Another direction in engineered ACT technology is using natural killer (NK) cells as an alternative to T cells. NK cells have been proved to be safer in terms of CRS and GVHD risks than modified T cells with insensitivity to MHC and the presence of inhibitory receptor as a safety switch (67). A phase I study using mesothelin specific CAR-NK cells to treat epithelial ovarian cancer is ongoing (NCT03692637).

## Summary

Engineered T cells therapy for gynecologic cancer would inevitably face the existence of practical challenges such as safety concerns, difficult choices of appropriate antigen, the immunosuppressive tumor microenvironment, the short pharmacological duration and high finical cost. Based on a substantial number of preclinical researches with various models, series of phase I/II clinical trials are exploring the optimal route and dosage of ACT products, or whether a combination with surgery, radiotherapy, chemotherapy, or other immunotherapies would facilitate the treatment of malignant gynecologic tumors with decreased recurrence and metastasis rate, reduced adverse drug reactions, and improved life quality of patients.

## Author Contributions

All authors listed have made a substantial, direct, and intellectual contribution to the work and approved it for publication.

## Fundings

This work was supported by the National Natural Science Foundation of China [Grant nos. 82073165]; the Beijing Kanghua Traditional Chinese and Western Medicine Development Fund [Grant nos.KH-2020-LJJ-043]; and the Provincial College Student Innovation and Entrepreneurship Training Program [S202012121060].

## Conflict of Interest

The authors declare that the research was conducted in the absence of any commercial or financial relationships that could be construed as a potential conflict of interest.

## Publisher’s Note

All claims expressed in this article are solely those of the authors and do not necessarily represent those of their affiliated organizations, or those of the publisher, the editors and the reviewers. Any product that may be evaluated in this article, or claim that may be made by its manufacturer, is not guaranteed or endorsed by the publisher.

## References

[1] Brentjens RJ Latouche JB Santos E Marti F Gong MC Lyddane C. Eradication of Systemic B-Cell Tumors by Genetically Targeted Human T Lymphocytes Co-Stimulated by CD80 and Interleukin-15. Nat Med (2003) 9:279–86. 10.1038/nm827 12579196

[2] Porter DL Hwang W Frey NV Lacey SF Shaw PA Loren AW. Chimeric Antigen Receptor T Cells Persist and Induce Sustained Remissions in Relapsed Refractory Chronic Lymphocytic Leukemia. Sci Transl Med (2015) 7:303ra139. 10.1126/scitranslmed.aac5415 PMC5909068 26333935

[3] Turtle CJ Hanafi LA Berger C Hudecek M Pender B Robinson E. Immunotherapy of Non-Hodgkin’s Lymphoma With a Defined Ratio of CD8+ and CD4+ CD19-Specific Chimeric Antigen Receptor-Modified T Cells. Sci Transl Med (2016) 8:355ra116. 10.1126/scitranslmed.aaf8621 PMC5045301 27605551

[4] Kochenderfer JN Dudley ME Kassim SH Somerville RPT Carpenter RO Stetler-Stevenson M. Chemotherapy-Refractory Diffuse Large B-Cell Lymphoma and Indolent B-Cell Malignancies can be Effectively Treated With Autologous T Cells Expressing an Anti-CD19 Chimeric Antigen Receptor. J Clin Oncol (2015) 33:540–9. 10.1200/jco.2014.56.2025 PMC4322257 25154820

[5] Turtle CJ Hay KA Hanafi LA Li D Cherian S Chen X. Durable Molecular Remissions in Chronic Lymphocytic Leukemia Treated With CD19-Specific Chimeric Antigen Receptor-Modified T Cells After Failure of Ibrutinib. J Clin Oncol (2017) 35:3010–20. 10.1200/jco.2017 PMC5590803 28715249

[6] Broderick JM. FDA Grants LN-145 Breakthrough Designation for Cervical Cancer (2019). Available at: https://www.onclive.com/web-exclusives/fda-grants-ln145-breakthrough-designation-for-cervical-cancer (Accessed on January 2020).

[7] Jazaeri AA Zsiros E Amaria RN Artz AS Edwards RP Wenham RM. Safety and Efficacy of Adoptive Cell Transfer Using Autologous Tumor Infiltrating Lymphocytes (LN-145) for Treatment of Recurrent, Metastatic, or Persistent Cervical Carcinoma. J Clin Oncol (2019) 37:2538. 10.1200/jco.2019.37.15_suppl.2538

[8] Bouchkouj N Kasamon YL de Claro RA George B Lin X Lee S. FDA Approval Summary: Axicabtagene Ciloleucel for Relapsed or Refractory Large B-Cell Lymphoma. Clin Cancer Res (2019) 25:1702–8. 10.1158/1078-0432.ccr-18-2743 30413526

[9] O’Leary MC Lu X Huang Y Lin X Mahmood I Przepiorka D. FDA Approval Summary: Tisagenlecleucel for Treatment of Patients With Relapsed or Refractory B-Cell Precursor Acute Lymphoblastic Leukemia. Clin Cancer Res (2019) 25:1142–6. 10.1158/1078-0432.ccr-18-2035 30309857

[10] Morgan RA Dudley ME Wunderlich JR Hughes MS Yang JC Sherry RM. Cancer Regression in Patients After Transfer of Genetically Engineered Lymphocytes. Science (2006) 314:126–9. 10.1126/science.1129003 PMC2267026 16946036

[11] Kochenderfer JN Wilson WH Janik JE Dudley ME Stetler-Stevenson M Feldman SA. Eradication of B-Lineage Cells and Regression of Lymphoma in a Patient Treated With Autologous T Cells Genetically Engineered to Recognize CD19. Blood (2010) 116:4099–102. 10.1182/blood-2010-04-281931 PMC2993617 20668228

[12] Chamie K Lee JH Rock A Rhode PR Soon-Shiong P. Preliminary Phase 2 Clinical Results of IL-15rαfc Superagonist N-803 With BCG in BCG-Unresponsive non-Muscle Invasive Bladder Cancer (NMIBC) Patients. J Clin Oncol (2019) 37:4561–1. 10.1200/jco.2019.37.15_suppl.4561

[13] Deniger DC Pasetto A Tran E Parkhurst MR Cohen CJ Robbins PF. Stable, Nonviral Expression of Mutated Tumor Neoantigen-Specific T-Cell Receptors Using the Sleeping Beauty Transposon/Transposase System. Mol Ther (2016) 24:1078–89. 10.1038/mt.2016.51 PMC4923320 26945006

[14] Jiang X Xu J Liu M Xing H Wang Z Huang L. Adoptive CD8(+) T Cell Therapy Against Cancer: Challenges and Opportunities. Cancer Lett (2019) 462:23–32. 10.1016/j.canlet.2019.07.017 31356845

[15] Eshhar Z Waks T Gross G Schindler DG. Specific Activation and Targeting of Cytotoxic Lymphocytes Through Chimeric Single Chains Consisting of Antibody-Binding Domains and the Y or C Subunits of the Immunoglobulin and T-Cell Receptors. Proc Natl Acad Sci USA (1993) 90:720–4. 10.1073/pnas.90.2.720 PMC45737 8421711

[16] Lai Y Wen J Wei X Qin L Lai P Zhao R. Toll-Like Receptor 2 Costimulation Potentiates the Antitumor Efficacy of CAR T Cells. Leukemia (2017) 32:801–8. 10.1038/leu.2017.249 28841215

[17] Chmielewski M Abken H. TRUCKs: The Fourth Generation of CARs. Expert Opin Biol Th (2015) 15:1145–54. 10.1517/14712598.2015.1046430 25985798

[18] Wang M Munoz J Goy A Locke FL Jacobson CA Hill BT. KTE-X19 CAR T-Cell Therapy in Relapsed or Refractory Mantle-Cell Lymphoma. N Engl J Med (2020) 382:1331–42. 10.1056/NEJMoa1914347 PMC7731441 32242358

[19] Abramson JS Palomba ML Gordon LI Lunning MA Wang M Arnason J. Lisocabtagene Maraleucel for Patients With Relapsed or Refractory Large B-Cell Lymphomas (TRANSCEND NHL 001): A Multicentre Seamless Design Study. Lancet (2020) 396:839–52. 10.1016/S0140-6736(20)31366-0 32888407

[20] Munshi NC Anderson LD Shah N Jagannath S Berdeja JG Lonial S. Idecabtagene Vicleucel (Ide-Cel; Bb2121), a BCMA-Targeted CAR T-Cell Therapy, in Patients With Relapsed and Refractory Multiple Myeloma (RRMM): Initial KarMMa Results. J Clin Oncol (2020) 38:8503–3. 10.1200/jco.2020.38.15_suppl.8503

[21] Kandalaft LE Odunsi K Coukos G. Immunotherapy in Ovarian Cancer: Are We There Yet? J Clin Oncol (2019) 37:2460–71. 10.1200/jco.19.00508 31403857

[22] Kerkar SP Wang Z Lasota J Park T Patel K Groh E. MAGE-A is More Highly Expressed Than NY-ESO-1 in a Systematic Immunohistochemical Analysis of 3668 Cases. J Immunother (2016) 39:181–7. 10.1097/cji.0000000000000119 PMC4831141 27070449

[23] Hong DS Van Tine BA Olszanski AJ Johnson ML Liebner DA Trivedi T. Phase I Dose Escalation and Expansion Trial to Assess the Safety and Efficacy of ADP-A2M4 SPEAR T Cells in Advanced Solid Tumors. J Clin Ocnol (2020) 38:102. 10.1200/jco.2020.38.15_suppl.102

[24] Anderson VE Weber AM Wiedermann GE Pachnio A Dauleh S Ahmed T. Enhanced Activity of Second-Generation MAGE-A4 SPEAR T-Cells Through Co-Expression of a CD8α Homodimer. Proceedings: AACR Annu Meeting (2019) 79:2313. 10.1158/1538-7445.am2019-2313

[25] Butler MO Sotov V Saibil S Bonilla L Boross-Harmer S Fyrsta M. 1183pd-Adoptive T Cell Therapy With TBI-1301 Results in Gene-Engineered T Cell Persistence and Anti-Tumour Responses in Patients With NY-ESO-1 Expressing Solid Tumours. Ann Oncl (2019) 30:v481. 10.1093/annonc/mdz253.009

[26] Anderson KG Voillet V Bates BM Chiu EY Burnett MG Garcia NM. Engineered Adoptive T-Cell Therapy Prolongs Survival in a Preclinical Model of Advanced-Stage Ovarian Cancer. Cancer Immuno Res (2019) 7:1412–25. 10.1158/2326-6066.cir-19-0258 PMC6726582 31337659

[27] Haas AR Tanyi JL O Hara MH Gladney WL Lacey SF Torigian DA. Phase I Study of Lentiviral-Transduced Chimeric Antigen Receptor-Modified T Cells Recognizing Mesothelin in Advanced Solid Cancers. Mol Ther (2019) 27:1919–29. 10.1016/j.ymthe.2019.07.015 PMC6838875 31420241

[28] Fang J Ding N Guo X Sun Y Zhang Z Xie B. αpd-1-mesoCAR-T Cells Partially Inhibit the Growth of Advanced/Refractory Ovarian Cancer in a Patient Along With Daily Apatinib. J Immunother Cancer (2021) 9:e001162. 10.1136/jitc-2020-001162 33589520 PMC7887368

[29] Zhao Y Liu Z Wang X Wu H Zhang J Yang J. Treatment With Humanized Selective CD19CAR-T Cells Shows Efficacy in Highly Treated B-ALL Patients Who Have Relapsed After Receiving Murine-Based CD19CAR-T Therapies. Clin Cancer Res (2019) 25:5595–607. 10.1158/1078-0432.ccr-19-0916 31300451

[30] Koneru M Cearbhaill R O Pendharkar S Spriggs DR Brentjens RJ. A Phase I Clinical Trial of Adoptive T Cell Therapy Using IL-12 Secreting MUC-16ecto Directed Chimeric Antigen Receptors for Recurrent Ovarian Cancer. J Transl Med (2015) 13:102. 10.1186/s12967-015-0460-x 25890361 PMC4438636

[31] Boylan KL Buchanan PC Manion RD Shukla DM Braumberger K Bruggemeyer C. The Expression of Nectin-4 on the Surface of Ovarian Cancer Cells Alters Their Ability to Adhere, Migrate, Aggregate, and Proliferate. Oncotarget (2017) 8:9717–38. 10.18632/oncotarget.14206 PMC5354766 28038455

[32] Rodriguez-Garcia A Sharma P Poussin M Boesteanu AC Minutolo NG Gitto SB. CAR T Cells Targeting MISIIR for the Treatment of Ovarian Cancer and Other Gynecologic Malignancies. Mol Ther (2020) 28:548–60. 10.1016/j.ymthe.2019.11.028 PMC7001088 31870622

[33] Chan T Chakiath M Shepard L Metenou S Carvajal-Borda F Velez J. Abstract 6593: PRGN-3005 UltraCAR-T™: Multigenic CAR-T Cells Generated Using non-Viral Gene Delivery and Rapid Manufacturing Process for the Treatment of Ovarian Cancer. Cancer Res (2020) 80:6593. 10.1158/1538-7445.am2020-6593

[34] Naumann RW Hollebecque A Meyer T Devlin M Oaknin A Kerger J. Safety and Efficacy of Nivolumab Monotherapy in Recurrent or Metastatic Cervical, Vaginal, or Vulvar Carcinoma: Results From the Phase I/II Checkmate 358 Trial. J Clin Oncol (2019) 37:2825–34. 10.1200/jco.19.00739 PMC6823884 31487218

[35] Bray F Ferlay J Soerjomataram I Siegel RL Torre LA Jemal A. Global Cancer Statistics 2018: GLOBOCAN Estimates of Incidence and Mortality Worldwide for 36 Cancers in 185 Countries. CA-Cancer J Clin (2018) 68:394–424. 10.3322/caac.21492 30207593

[36] Draper LM Kwong MLM Gros A Stevanović S Tran E Kerkar S. Targeting of HPV-16+ Epithelial Cancer Cells by TCR Gene Engineered T Cells Directed Against E6. Clin Cancer Res (2015) 21:4431–9. 10.1158/1078-0432.ccr-14-3341 PMC4603283 26429982

[37] Jin BY Campbell TE Draper LM Stevanovic S Weissbrich B Yu Z. Engineered T Cells Targeting E7 Mediate Regression of Human Papillomavirus Cancers in a Murine Model. JCI Insight (2018) 3:e99488. 10.1172/jci.insight.99488 PMC5931134 29669936

[38] Doran SL Stevanovic S Adhikary S Gartner JJ Jia L Kwong MLM. T-Cell Receptor Gene Therapy for Human Papillomavirus-Associated Epithelial Cancers: A First-in-Human, Phase I/II Study. J Clin Oncol (2019) 37:2759–68. 10.1200/jco.18 PMC6800280 31408414

[39] Nagarsheth NB Norberg SM Sinkoe AL Adhikary S Meyer TJ Lack JB. TCR-Engineered T Cells Targeting E7 for Patients With Metastatic HPV-Associated Epithelial Cancers. Nat Med (2021) 27:1–7. 10.1038/s41591-020-01225-1 33558725 PMC9620481

[40] Bryson P Jia Q Chen G Li S Fang J Zhao L. 1227p-HPV16 E6-Specific TCR-T Armored With Checkpoint Blockade in the Treatment of Cervical Cancer. J Immunother Cancer (2019) 30:v502. 10.1093/annonc/mdz253.053

[41] Golubovskaya V Berahovich R Zhou H Xu S Harto H Li L. CD47-CAR-T Cells Effectively Kill Target Cancer Cells and Block Pancreatic Tumor Growth. Cancers (2017) 9:139. 10.3390/cancers9100139 PMC5664078 29065481

[42] He Y Li X Yin C Wu Y. Killing Cervical Cancer Cells by Specific Chimeric Antigen Receptor-Modified T Cells. J Reprod Immunol (2020) 139:103115. 10.1016/j.jri.2020.103115 32199196

[43] Zhang Y Li X Zhang J Mao L. Novel Cellular Immunotherapy Using NKG2D CAR-T for the Treatment of Cervical Cancer. BioMed Pharmacother (2020) 131:110562. 10.1016/j.biopha.2020.110562 32920508

[44] Sung H Ferlay J Siegel RL Laversanne M Soerjomataram I Jemal A. Global Cancer Statistics 2020: GLOBOCAN Estimates of Incidence and Mortality Worldwide for 36 Cancers in 185 Countries. CA: Cancer J Clin (2021) 0:1–41. 10.3322/caac.21660 33538338

[45] Siegel RL Miller KD Jemal A. Cancer Statistics, 2019. CA: A Cancer J Clin (2019) 69:7–34. 10.3322/caac.21551 30620402

[46] Singh N Gilks CB. Vulval Squamous Cell Carcinoma and Its Precursors. Histopathology (2019) 76:128–38. 10.1111/his.13989 31846523

[47] Morgan RA Chinnasamy N Abate-Daga D Gros A Robbins PF Zheng Z. Cancer Regression and Neurological Toxicity Following Anti-MAGE-A3 TCR Gene Therapy. J Immunother (2013) 36:133–51. 10.1097/cji.0b013e3182829903 PMC3581823 23377668

[48] Cameron BJ Gerry AB Dukes J Harper JV Kannan V Bianchi FC. Identification of a Titin-Derived HLA-A1-Presented Peptide as a Cross-Reactive Target for Engineered MAGE A3-Directed T Cells. Sci Transl Med (2013) 5:197ra103. 10.1126/scitranslmed.3006034 PMC6002776 23926201

[49] Linette GP Stadtmauer EA Maus MV Rapoport AP Levine BL Emery L. Cardiovascular Toxicity and Titin Cross-Reactivity of Affinity-Enhanced T Cells in Myeloma and Melanoma. Blood (2013) 122:863–71. 10.1182/blood-2013-03-490565 PMC3743463 23770775

[50] Matsuda T Leisegang M Park J Ren L Kato T Ikeda Y. Induction of Neoantigen-Specific Cytotoxic T Cells and Construction of T-Cell Receptor-Engineered T Cells for Ovarian Cancer. Clin Cancer Res (2018) 24:5357–67. 10.1158/1078-0432.ccr-18-0142 29720506

[51] Neelapu SS Locke FL Bartlett NL Lekakis L Miklos D Jacobson CA. Kte-C19 (Anti-CD19 CAR T Cells) Induces Complete Remissions in Patients With Refractory Diffuse Large B-Cell Lymphoma (DLBCL): Results From the Pivotal Phase 2 Zuma-1. Blood (2016) 128:LBA–6. 10.1182/blood.V128.22.LBA-6.LBA-6

[52] Frey N Levine B Lacey S Grupp S Maude S Schuster S. Refractory Cytokine Release Syndrome in Recipients of Chimeric Antigen Receptor (CAR) T Cells. Blood (2014) 124:2296. 10.1182/blood.V124.21.2296.2296

[53] Shimabukuro-Vornhagen A Gödel P Subklewe M Stemmler HJ Schlößer HA Schlaak M. Cytokine Release Syndrome. J Immunother Cancer (2018) 6:56. 10.1186/s40425-018-0343-9 29907163 PMC6003181

[54] Ruella M Barrett DM Kenderian SS Shestova O Hofmann TJ Perazzelli J. Dual CD19 and CD123 Targeting Prevents Antigen-Loss Relapses After CD19-Directed Immunotherapies. J Clin Invest (2016) 126:3814–26. 10.1172/jci87366 PMC5096828 27571406

[55] Ghoneum A Afify H Salih Z Kelly M Said N. Role of Tumor Microenvironment in the Pathobiology of Ovarian Cancer: Insights and Therapeutic Opportunities. Cancer Med-Us (2018) 7:5047–56. 10.1002/cam4.1741 PMC6198242 30133163

[56] Adachi K Kano Y Nagai T Okuyama N Sakoda Y Tamada K. IL-7 and CCL19 Expression in CAR-T Cells Improves Immune Cell Infiltration and CAR-T Cell Survival in the Tumor. Nat Biotechnol (2018) 36:346–51. 10.1038/nbt.4086 29505028

[57] Craddock JA Lu A Bear A Pule M Brenner MK Rooney CM. Enhanced Tumor Trafficking of GD2 Chimeric Antigen Receptor T Cells by Expression of the Chemokine Receptor CCR2b. J Immunother (2010) 33:780–8. 10.1097/CJI.0b013e3181ee6675 PMC2998197 20842059

[58] Di Stasi A De Angelis B Rooney CM Zhang L Mahendravada A Foster AE. T Lymphocytes Coexpressing CCR4 and a Chimeric Antigen Receptor Targeting CD30 Have Improved Homing and Antitumor Activity in a Hodgkin Tumor Model. Blood (2009) 113:6392–402. 10.1182/blood-2009-03-209650 PMC2710932 19377047

[59] Zhang F Stephan SB Ene CI Smith TT Holland EC Stephan MT. Nanoparticles That Reshape the Tumor Milieu Create a Therapeutic Window for Effective T Cell Therapy in Solid Malignancies. Cancer Res (2018) 78:306–2018. 10.1158/0008-5472.can-18-0306 PMC6030470 29760047

[60] Wu S Zhu W Peng Y Wang L Hong Y Huang L. The Antitumor Effects of Vaccine-Activated CD8(+) T Cells Associate With Weak TCR Signaling and Induction of Stem-Like Memory T Cells. Cancer Immunol Res (2017) 5:908–19. 10.1158/2326-6066.cir-17-0016 PMC5626646 28851693

[61] Presotto D Erdes E Duong MN Allard M Regamey P Quadroni M. Fine-Tuning of Optimal TCR Signaling in Tumor-Redirected CD8 T Cells by Distinct TCR Affinity-Mediated Mechanisms. Front Immunol (2017) 8:1564. 10.3389/fimmu.2017.01564 29187853 PMC5694758

[62] Abdelsamed HA Moustaki A Fan Y Dogra P Ghoneim HE Zebley CC. Human Memory CD8 T Cell Effector Potential Is Epigenetically Preserved During *In Vivo* Homeostasis. J Exp Med (2017) 214:593–1606. 10.1084/jem.20161760 PMC5461005 28490440

[63] Blaeschke F Stenger D Kaeuferle T Willier S Lotfi R Kaiser AD. Induction of a Central Memory and Stem Cell Memory Phenotype in Functionally Active CD4+ and CD8+ CAR T Cells Produced in an Automated Good Manufacturing Practice System for the Treatment of CD19+ Acute Lymphoblastic Leukemia. Cancer Immunol Immun (2018) 67:1053–66. 10.1007/s00262-018-2155-7 PMC11028239 29605883

[64] Roddie C O’Reilly M Dias Alves Pinto J Vispute K Lowdell M. Manufacturing Chimeric Antigen Receptor T Cells: Issues and Challenges. Cytotherapy (2019) 21:327–40. 10.1016/j.jcyt.2018.11.009 30685216

[65] Depil S Duchateau P Grupp SA Mufti G and Poirot L. ‘Off- the-Shelf’ Allogeneic CAR T Cells: Development and Challenges. Nat Rev Drug Discov (2020) 19:185–99. 10.1038/s41573-019-0051-2 31900462

[66] Ren J Liu X Fang C Jiang S June CH Zhao Y. Multiplex Genome Editing to Generate Universal CAR T Cells Resistant to PD1 Inhibition. Clin Cancer Res (2017) 23:2255–66. 10.1158/1078-0432.ccr-16-1300 PMC5413401 27815355

[67] Zhang C Oberoi P Oelsner S Waldmann A Lindner A Tonn T. Chimeric Antigen Receptor-Engineered NK-92 Cells: An Off-The-Shelf Cellular Therapeutic for Targeted Elimination of Cancer Cells and Induction of Protective Antitumor Immunity. Front Immunol (2017) 8:533. 10.3389/fimmu.2017.00533 28572802 PMC5435757

